# HCMV Infection of Human Trophoblast Progenitor Cells of the Placenta Is Neutralized by a Human Monoclonal Antibody to Glycoprotein B and Not by Antibodies to the Pentamer Complex

**DOI:** 10.3390/v6031346

**Published:** 2014-03-19

**Authors:** Martin Zydek, Matthew Petitt, June Fang-Hoover, Barbara Adler, Lawrence M. Kauvar, Lenore Pereira, Takako Tabata

**Affiliations:** 1 Department of Cell and Tissue Biology, University of California San Francisco, 513 Parnassus Avenue, San Francisco, CA 94143, USA; E-Mails: martin.zydek@ucsf.edu (M.Z.); matthew.petitt@ucsf.edu (M.P.); june.fang-hoover@ucsf.edu (J.F.-H.); takako.tabata@ucsf.edu (T.T.); 2 Division of Virology, Max von Pettenkofer-Institute, Ludwig-Maximilians-University Munich, Pettenkoferstr. 9A, D-80336 Munich, Germany; E-Mail: adler_b@mvp.uni-muenchen.de; 3 Trellis Bioscience, LLC, 2-B Corporate Drive, South San Francisco, CA 94080, USA; E-Mail: lkauvar@trellisbio.com

**Keywords:** HCMV, congenital, trophoblast, progenitors, neutralizing, placenta, development, neutralization, pentamer, hyperimmune globulin

## Abstract

Human cytomegalovirus (HCMV) is the major viral cause of congenital infection and birth defects. Primary maternal infection often results in virus transmission, and symptomatic babies can have permanent neurological deficiencies and deafness. Congenital infection can also lead to intrauterine growth restriction, a defect in placental transport. HCMV replicates in primary cytotrophoblasts (CTBs), the specialized cells of the placenta, and inhibits differentiation/invasion. Human trophoblast progenitor cells (TBPCs) give rise to the mature cell types of the chorionic villi, CTBs and multi-nucleated syncytiotrophoblasts (STBs). Here we report that TBPCs are fully permissive for pathogenic and attenuated HCMV strains. Studies with a mutant virus lacking a functional pentamer complex (gH/gL/pUL128-131A) showed that virion entry into TBPCs is independent of the pentamer. In addition, infection is blocked by a potent human neutralizing monoclonal antibody (mAb), TRL345, reactive with glycoprotein B (gB), but not mAbs to the pentamer proteins pUL130/pUL131A. Functional studies revealed that neutralization of infection preserved the capacity of TBPCs to differentiate and assemble into trophospheres composed of CTBs and STBs *in vitro*. Our results indicate that mAbs to gB protect trophoblast progenitors of the placenta and could be included in antibody treatments developed to suppress congenital infection and prevent disease.

## 1. Introduction

Human cytomegalovirus (HCMV) is the major viral cause of congenital infections and affects 1%–3% of live births in the U.S. [1]. Virus transmission to the fetus occurs in 40%–50% of cases of primary infection and can result in birth defects, including severe neurological disorders, impaired hearing, vision loss and intrauterine growth restriction (IUGR), a defect that results from compromised placental function [2,3,4]. In contrast, due to immunity from maternal antibodies, recurrent infection is associated with a reduced viral transmission rate (0.1% to 2%) [5]. Although recent studies indicate that the frequency of hearing loss is similar in pregnancies complicated by primary and recurrent infection, the severity is greater in cases of primary infection [6,7].

Currently, there are no approved therapeutics for congenital HCMV infection in cases of primary maternal infection due to concerns over the toxicity and teratogenicity of available antiviral drugs (reviewed in [8,9]). A recently developed therapeutic approach is the administration of hyperimmune globulin (HIG), a pooled immunoglobulin preparation from donors with high-avidity anti-HCMV antibodies. In initial (non-randomized) clinical studies, treatment of pregnant women with HIG within 4 to 6 weeks after seroconversion was found to improve fetal outcome [10,11,12,13]. Analysis of placentas revealed that viral replication was reduced and compensatory development enabled growth of chorionic villi increasing the placental surface perfused by maternal blood [12,14]. However, reported studies were small and the results should be confirmed by double-blinded clinical trials [15]. To date, administration of HIG has been approved by the Food and Drug Administration only for transplant recipients [16,17,18]. As promising novel therapeutics, human monoclonal antibodies (mAbs) specific for epitopes on HCMV proteins have been developed using technological advancement of human B-cell cloning from highly seropositive donors [19,20,21,22,23].

Chorionic villi, the functional units of the placenta, are composed of a stromal core that contains blood vessels continuous with the fetal vasculature. The core is surrounded by a basement membrane populated by villus cytotrophoblasts (vCTBs), covered by a multinucleated syncytiotrophoblast (STB) layer. At the tips of the villi, vCTBs differentiate and switch their adhesion phenotype to that of invasive cytotrophoblasts (iCTBs), which penetrate the decidua and remodel uterine arteries, providing maternal blood to the placenta [24,25]. STBs, which are bathed in maternal blood, mediate transport of nutrients and oxygen to the fetal bloodstream. In contrast to CTBs, whose proliferative potential is limited [26,27], trophoblast progenitor cells (TBPCs), resident in the chorion, proliferate and differentiate into the mature trophoblast populations [28].

Previous studies from our lab suggested that during the course of transmission, HCMV first replicates in uterine blood vessels, then spreads to differentiating iCTBs, where infection downregulates cell-matrix adhesion molecules and impairs cell differentiation/invasion [29,30,31,32,33]. Studies of congenital infection in placentas from early gestation and at term indicated a central role for high-avidity, neutralizing antibodies in suppression of infection [34,35,36]. We recently reported that some cases of IUGR have underlying primary or recurrent maternal infection, which impaired placental development [4]. In a case of IUGR associated with preterm delivery, chorionic villi contained unusual clusters of extravillous, cytokeratin 7 (CK7)-positive CTBs, suggesting arrested differentiation. In cases of symptomatic congenital infection, infected cell proteins were detected in TBPCs in the chorion, suggesting that virus replication could interfere with TBPC self-renewal and differentiation [37].

HCMV encodes several glycoprotein complexes in the virion envelope that function in attachment and penetration of host cells. A highly studied envelope protein important for virion entry into all cell types is gB [38,39]. gB facilitates attachment to the cell surface by binding to heparin sulfate glycosaminoglycans [40,41], cellular receptors [42,43] and integrins [44,45] and, in cooperation with gH/gL complexes, mediates membrane fusion [46]. In contrast to the fibroblast-adapted (attenuated) strains AD169 and Towne, clinical strains, such as VR1814 and TB40/E, are pathogenic and express the pentamer glycoprotein complex gH/gL/pUL128-131A, which is required for virus entry into epithelial and endothelial cells [47,48] but not fibroblasts [49,50]. AD169 and Towne have acquired mutations in the UL128-131A locus, lack a functional pentamer and are impaired in infection of epithelial and endothelial cells [51,52].

Recently, TBPCs were isolated from the chorions of human placentas, and lines of continuously self-renewing cells were established [27]. These TBPCs express the trophoblast marker CK7, factors for stem cell self-renewal, including HMGA2 (high-mobility group AT-hook 2), and proteins required for trophoblast fate specification, GATA-3, GATA-4, Eomes (Eomesodermin) and GCM-1 (glial cell missing homolog 1). When cultured under differentiation conditions, TBPCs aggregate and form spheres (trophospheres) that upregulate HLA-G, a marker of differentiating iCTBs, and increase expression of human placental lactogen and human chorionic gonadotropin (normally secreted from STBs) [27], in accord with differentiation *in utero*.

Here, we have identified glycoproteins involved in entry of HCMV virions into TBPCs established from first and second trimester placentas and measured the extent to which neutralizing mAbs to gB and the pentameric complex block infection. We found that TBPCs are susceptible to infection with both the pathogenic VR1814 and the attenuated AD169 strain and that both strains undergo lytic viral replication in TBPCs. In addition, we found that virion entry into TBPCs was independent of the pentamer complex, as shown by infection with a UL131A-deficient mutant [53,54] and confirmed by failure of anti-pentamer mAbs to block VR1814 infection. Importantly, virion entry was found to depend on gB function. Consequently, neutralization of infection with an anti-gB mAb (TRL345) was found to preserve the ability of TBPCs to differentiate and form trophospheres.

## 2. Results and Discussion

### 2.1. TBPCs from First and Second Trimesters Are Permissive for Pathogenic and Attenuated HCMV Strains

**Figure 1:**
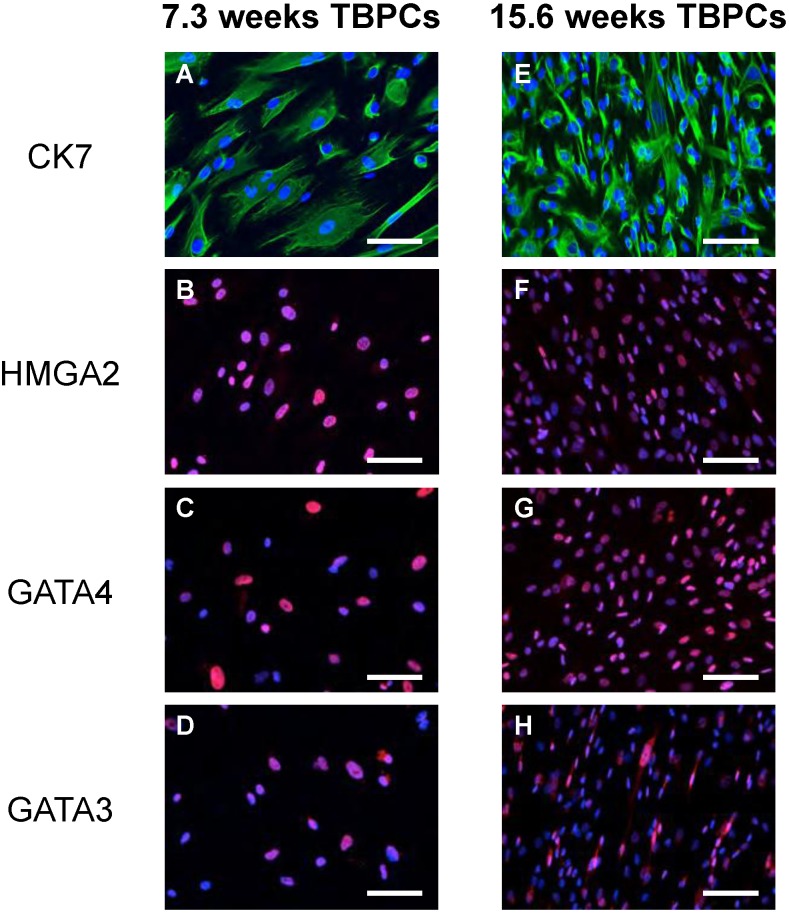
TBPCs express markers of trophoblasts and pluripotency. TBPCs from 7.3 weeks (left panel) and 15.6 weeks (right panel) of gestation were immunostained for CK7 (**A**, **E**), HMGA2 (**B**, **F**), GATA-4 (**C**, **G**) or GATA-3 (**D**, **H**). Nuclei were counterstained with DAPI. Scale bar = 100 µm.

TBPC expression of markers for trophoblasts (CK7), stem cell self-renewal (HMGA2) and trophoblast fate acquisition (GATA-4 and GATA-3) was confirmed by immunostaining. Over 90% of TBPCs in culture expressed these markers (Figure 1).

**Figure 2:**
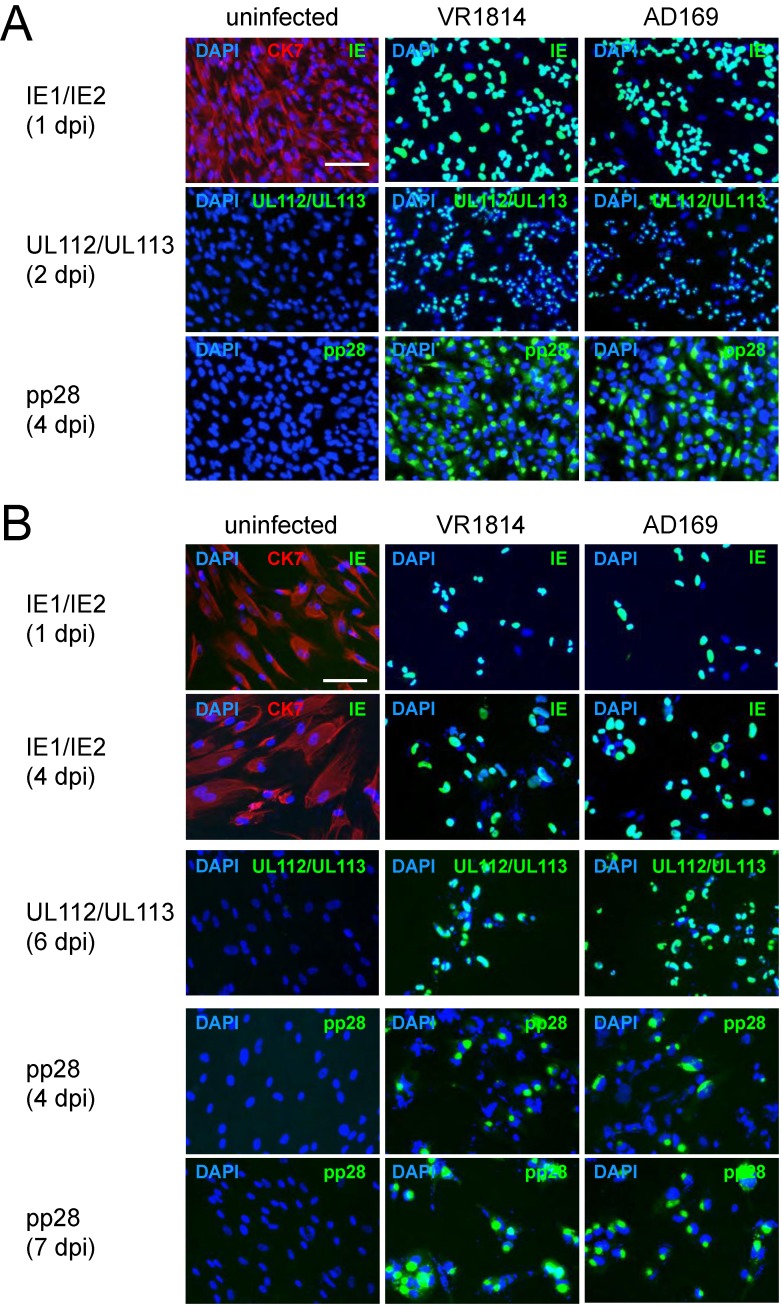
VR1814 and AD169 replicate in TBPCs. Cells from 15.6 weeks (**A**) or 7.3 weeks (**B**) of gestation were infected (MOI 2) and immunostained for HCMV IE1, pUL112/pUL113 or pp28 at the indicated time points (green). Mock-infected controls were immunostained for CK7 (red). Nuclei were counterstained with DAPI (blue). Scale bar = 100 µm.

To determine whether TBPCs isolated from placentas at 7.3 and 15.6 weeks of gestation were susceptible to infection, suspensions of VR1814 and AD169 were adsorbed to TBPCs and the cells immunostained at the indicated time points for expression of immediate-early protein IE1, early proteins pUL112/pUL113 and late protein pp28. We found that viral proteins were expressed at comparable levels in VR1814- and AD169-infected cells (Figure 2), indicating that TBPCs were susceptible to lytic HCMV infection. In 15.6 weeks TBPCs, nearly all cells expressed IE1 in nuclei at 1 day post infection (dpi), and pUL112/pUL113 were detected at 2 dpi. pp28 was present in most cells at 4 dpi (Figure 2A) suggesting viral DNA replication [55]. In 7.3 weeks TBPCs, fewer cells expressed viral proteins. Approximately 70% expressed IE1 at 1 dpi and 85% at 4 dpi (Figure 2B). pUL112/pUL113 and pp28 were expressed in nearly all cells by 7 dpi, suggesting delayed viral gene expression. Nonetheless, almost all TBPCs from both gestational ages expressed late infected-cell proteins within a week. Altogether, our results indicate that first and second trimester TBPCs are permissive for pathogenic and attenuated virus strains. Because results for first and second trimester TBPCs were similar, only TBPCs from 15.6 weeks of gestation were used for subsequent experiments.

### 2.2. HCMV-Infected TBPCs Produce Infectious Progeny Virions

**Figure 3:**
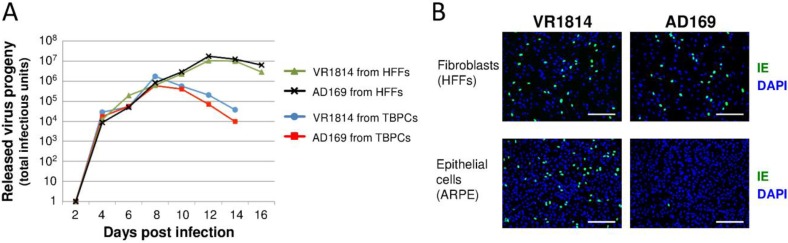
(**A**) Infected TBPCs release progeny virions. CM from VR1814- and AD169-infected TBPCs and HFFs (MOI 0.1) were collected at the indicated time points and titrated on HFFs. (**B**) Progeny virions from TBPCs retain tropism. CM from VR1814- and AD169-infected TBPCs (8 dpi) was added to HFFs and ARPE-19 cells, and IE1 was immunostained (1 dpi). Nuclei were counterstained with DAPI. Scale bar = 200 µm.

To determine if TBPCs were fully permissive and produced progeny virions, the cells were infected with either VR1814 or AD169, conditioned medium (CM) was collected at 48 h intervals, and viral progeny were quantified by titration on human foreskin fibroblasts (HFFs). For comparison of virus yield, HFFs were infected and analyzed in parallel. We found that relatively high levels of progeny virions were released from infected TBPCs (total ~10^6^ PFU per 2 cm^2^ plate on peak day), about 10- to 15-fold lower than the amount released from HFFs (Figure 3A). Consistent with the comparable levels of IE, early and late protein expression (Figure 2A), no significant differences were found between the levels of VR1814 and AD169 progeny released. The results indicate that, following virion entry into TBPCs, both pathogenic and attenuated HCMV strains complete the lytic infectious cycle.

Next, we determined whether viral progeny released from TBPCs retain tropism. CM harvested from VR1814- and AD169-infected TBPCs (8 dpi) were adsorbed to human retinal pigment epithelial cells (ARPE-19) or HFFs. Immunostaining for IE proteins showed that AD169 progeny failed to infect ARPE-19 cells (Figure 3B) whereas VR1814 progeny from infected TBPCs retained the ability to infect ARPE-19 and HFFs. These results suggest that progeny virions released from TBPCs in the infected chorion retain tropism and hence could spread to other placental cells—vCTBs, iCTBs, stromal fibroblasts in the villous core and endothelial cells in blood vessels—thereby promoting viral dissemination *in utero*.

### 2.3. Viral Entry into TBPCs Is Independent of the Pentamer Complex

**Figure 4:**
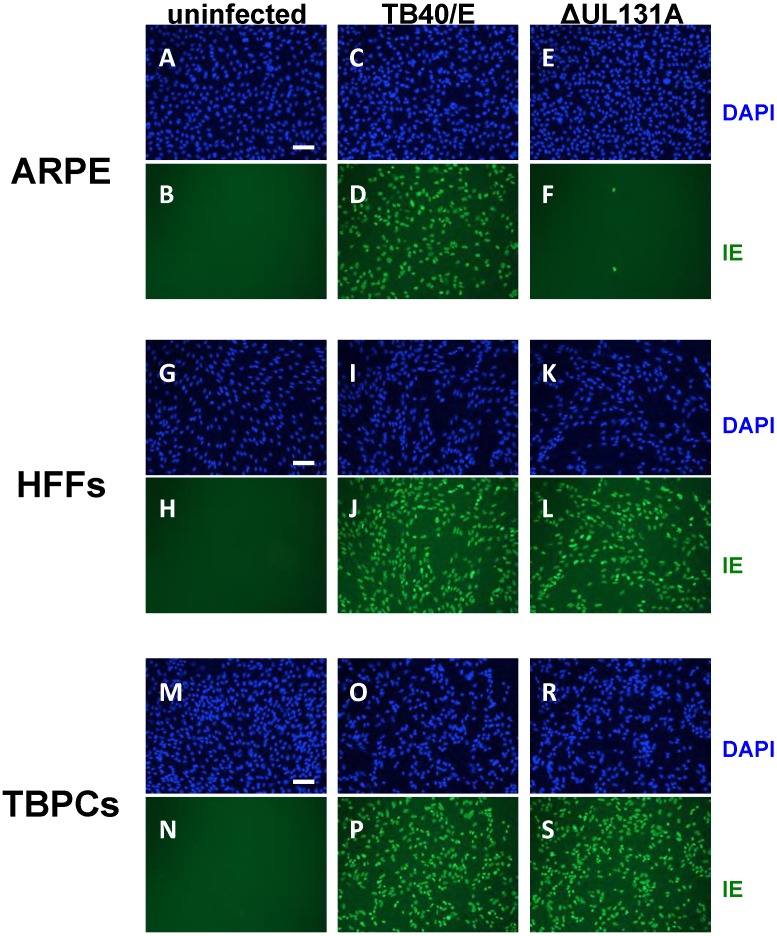
HCMV pentamer complex is dispensable for virion entry into TBPCs. ARPE-19 cells (**A**–**F**), HFFs (**G**–**L**) and TBPCs (**M**–**S**) were mock infected or infected (MOI 2) with parental TB40/E and UL131A-deficient mutant (ΔUL131A) and immunostained for IE1 proteins (2 dpi). Nuclei were counterstained with DAPI (blue). Scale bar = 100 µm.

The finding that TBPCs can be infected with AD169 suggested that the pentamer complex is not required for virion entry into these cells. To test this hypothesis, we performed experiments with the TB40/E parent virus and its UL131A-deficient mutant (ΔUL131A) [53]. The mutant has an early stop codon in the UL131A ORF, does not express a functional pentamer, and fails to enter epithelial and endothelial cells [53]. We confirmed the phenotype of parental TB40/E and ΔUL131A by infecting ARPE-19 cells and HFFs. As expected HFFs supported infection with both viruses as determined by IE protein expression at 2 dpi (Figure 4G–L), whereas ARPE-19 cells were only permissive for the parental virus TB40/E (Figure 4C,D), but not ΔUL131A (Figure 4E,F). To test TBPCs, we incubated the cells with TB40/E and ΔUL131A and found that in both cases >90% of cells expressed IE proteins at 2 dpi (Figure 4M–S). The results confirmed that the pentamer complex is dispensable for virion entry into TBPCs. These data also suggest that virion entry into TBPCs follows the pathway described for fibroblasts, which involves gB-mediated fusion at the plasma membrane at neutral pH [56], as opposed to the entry pathway into epithelial and endothelial cells, where virion uptake entails endocytosis [57,58].

### 2.4. TBPC Infection Is Blocked by mAbs to gB But Not mAbs to the Pentamer Complex

Unlike HIG preparations, human mAbs to viral glycoproteins are epitope specific and can have especially high affinity. Among the important HCMV proteins that have been targeted by human mAbs are gB [23] and components of the pentamer complex [19]. The finding that a viral mutant lacking a functional pentamer infects TBPCs at levels comparable to those of the parent, which expresses a functional pentamer, suggested that the pentamer is dispensable for virion entry. Consequently, we expected that mAbs that prevent HCMV entry into a broad range of cells (e.g., gB-specific mAbs) would block TBPC infection, whereas mAbs to the pentamer would not.

**Figure 5:**
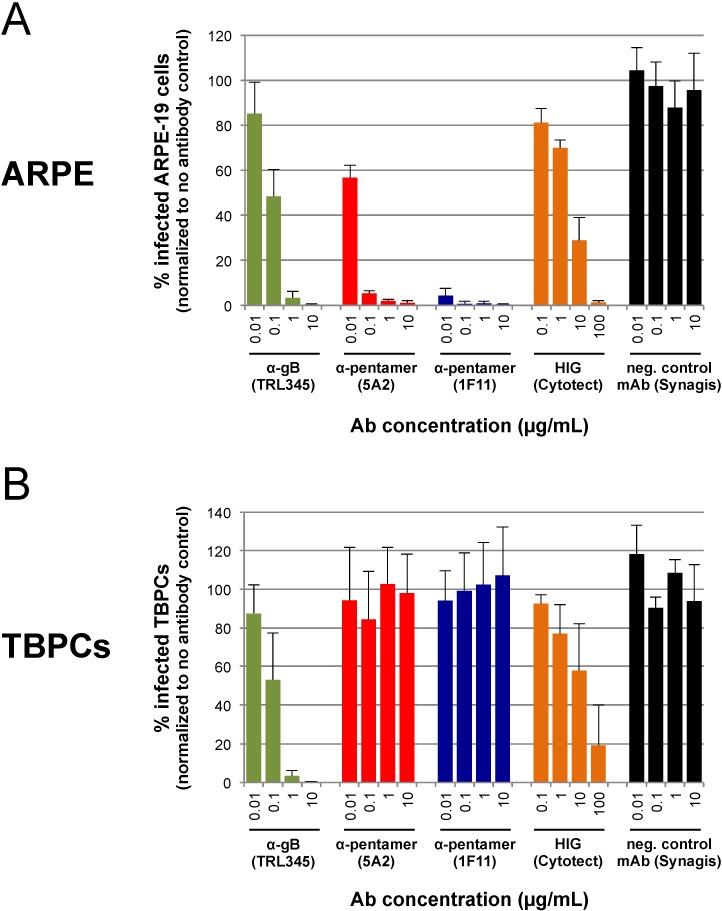
Infection of TBPCs is blocked by a mAb to gB but not mAbs to the pentamer complex. (**A**) VR1814 (MOI 0.01) was pre-incubated with medium alone, a serial log_10_ dilution of the indicated mAb (0.01–10 µg/mL) or HIG (0.1–100 µg/mL). Virus-antibody mixtures were then applied to ARPE-19 cells. At 2 dpi, IE1 was immunostained and numbers of infected cells were counted. Data were normalized to the no Ab control. Error bars indicate standard deviation. (**B**) TBPCs were incubated with virus-antibody mixtures and analyzed as described for panel **A**.

To test this hypothesis, we carried out neutralization assays with human mAbs to the pentamer component pUL130/pUL131A (mAbs 5A2 and 1F11) and a human mAb to gB (TRL345). These mAbs were selected by B-cell cloning and reported to have potent HCMV neutralizing titer in epithelial cells (anti‑pUL130/pUL131A and anti-gB) and fibroblasts (anti-gB) [19,23]. First, we verified that the mAbs and the HIG preparation blocked VR1814 infection of ARPE-19 cells in a dose-dependent manner (Figure 5). The control mAb (Synagis) lacked neutralizing activity (Figure 5A). Next, we found that mAbs to the pentamer (mAbs 5A2 and 1F11) failed to block VR1814 infection of TBPCs (Figure 5B), whereas anti-gB mAb TRL345 exhibited a dose-dependent neutralizing activity, almost completely blocking VR1814 infection (99% reduction) at 10 µg/mL (Figure 5B). HIG showed some neutralizing activity in TBPCs at high concentrations (~42% mean reduction at 10 µg/mL, ~81% at 100 µg/mL), but the efficiency was highly variable. It is difficult to compare HIG with HCMV neutralizing mAbs because the former is a polyclonal antibody mixture prepared from seropositive donors and contains mostly antibodies without antiviral activity. In contrast, each mAb recognizes a specific functional epitope on a single viral protein. Nonetheless, anti-gB mAb TRL345 blocked infection of TBPCs at 1 µg/mL to a greater extent than HIG at 100 µg/mL. These results indicate that a mAb to gB efficiently neutralizes HCMV infection of TBPCs as compared with HIG. 

### 2.5. Neutralization of VR1814 by Anti-gB mAb TRL345 Restores TBPC Differentiation

**Figure 6:**
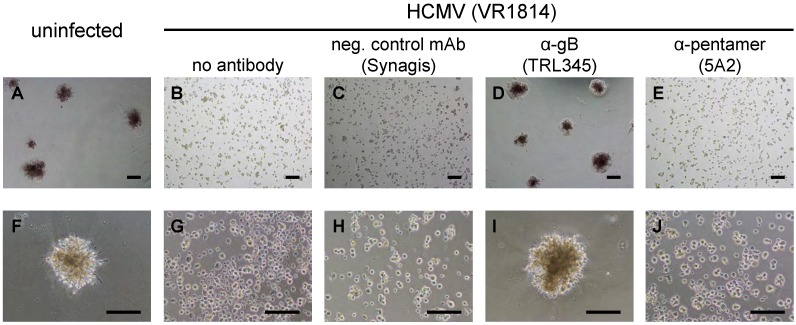
Neutralizing anti-gB mAb TRL345 precludes VR1814 infection and rescues TBPC differentiation. VR1814 was pretreated with 20 µg/mL of the indicated mAbs, and the mixtures were adsorbed to TBPCs. At 3 dpi, the cells were reseeded on matrigel and cultured in differentiation medium for 24 h followed by light microscopic analysis. Lower magnification photographs (upper panel), higher magnification photographs (lower panel). Scale bars = 200 µm.

When cultured under differentiation conditions, TBPCs form trophospheres and express markers of differentiated iCTBs and STBs [27], a process that mimics early differentiation *in utero*. In contrast, infected TBPCs fail to differentiate and form trophospheres [37]. We therefore examined whether TRL345-mediated virus neutralization could preserve the ability of TBPCs to differentiate and form trophospheres. For these experiments, TBPCs were infected with VR1814 or a virus-antibody mixture containing 20 µg/mL anti-gB mAb (TRL345), 20 µg/mL anti‑pentamer mAb (5A2) or 20 µg/mL negative control mAb (Synagis), or they were left uninfected. At 3 dpi, culture conditions were changed to induce differentiation (see Experimental Section). At 4 dpi, differentiating TBPCs (uninfected) formed trophospheres with diameters of approximately 200–250 µm (Figure 6A,F). In contrast, infected TBPCs generally failed to form spheres, and the few spheres that assembled had diameters of approximately 50 µm or less (Figure 6B,G). TBPCs incubated with anti-gB mAb-treated VR1814 retained the capacity to differentiate and formed large trophospheres (Figure 6D,I). In contrast, TBPCs incubated with VR1814 treated with mAbs to the pentamer or the negative control mAb failed to form large trophospheres (Figure 6C,E,H,J), consistent with the failure of pentamer-specific mAbs to block infection (Section 2.4). Thus, neutralizing anti-gB mAb TRL345 precludes HCMV infection of TBPCs and rescues differentiation.

### 2.6. Discussion

The studies presented here show that HCMV undergoes lytic replication in TBPCs and that viral entry into these cells depends on the functions of gB but not those of the pentamer complex. VR1814‑infected TBPCs release significant levels of progeny virions that retain epithelial cell tropism. These findings suggest infection of TBPCs could inhibit villous growth and differentiation and promote viral spread among various cell types in the placenta. Infection could decrease the population of TBPCs (Figure 2, Figure 3 and Figure 4) and impair differentiation (Figure 6), thereby reducing the mature trophoblast populations, CTBs and STBs.

These findings could also explain the observation that CTBs failed to differentiate into STBs in cases of IUGR with congenital HCMV infection, suggesting infection and paracrine effects could subvert placental development [4]. Passive immunization with HIG enables compensatory growth of chorionic villi, increasing STB surface area and exchange [14], suggesting that neutralizing antibodies reduce virus replication and improve outcome. HIG was reported to prevent virus transmission to the placenta and fetus when administrated soon after maternal seroconversion [10,11,12,13]. In accord with these findings, HIG reduces viral spread in the decidua in an *ex vivo* tissue model [59]. Animal models that simulate congenital infection are rare because of the unique anatomy and biology of the hematogenous human placenta. However, the guinea pig has been used to measure efficacy of guinea pig CMV (gpCMV) antibodies in reducing transplacental infection [60]. When pregnant guinea pigs were infected and passively immunized with gpCMV neutralizing antiserum, fetal survival increased significantly, and placental inflammation and IUGR were reduced [60]. Likewise, a gpCMV gB subunit vaccine elicited protective neutralizing antibodies in dams, resulting in lower rates of fetal infection and reduced pup mortality [61].

HCMV entry into fibroblasts requires gB and gH/gL [46,49,50], whereas entry into epithelial and endothelial cells requires gB and the pentamer complex gH/gL/pUL128-131A [47,48,49,50,51,62]. Consequently, anti-gB antibodies neutralize virus entry into all cell types, whereas antibodies to the pentamer components pUL128, pUL130 and pUL131A selectively block infection of epithelial and endothelial cells [19,63]. Interestingly, antibodies to the pentamer complex are the major active component of HIG [64], and delayed development of these antibodies correlates with transplacental transmission to the fetus [65]. Our results suggest that anti-pentamer mAbs may reduce but not prevent virus spread in the developing placenta, as they fail to protect TBPCs, and other cell types, including stromal fibroblasts from the villus core [29] and uterine smooth muscle cells (data not shown). Importantly, anti-gB mAbs, but not anti-pentamer mAbs, preserve the ability of TBPCs to differentiate. Moreover, we found that anti-gB mAb TRL345 had a higher efficiency and consistency as compared to HIG (Figure 5A,B). This high potency of mAb TRL345 is a consequence of the targeted, highly conserved AD-2 (Site I) epitope on gB [66], which the antibody binds to with high affinity [23]. While this neutralizing epitope is essential for gB function, it is poorly immunogenic, which explains why antibodies to this epitope are rare in human blood and consequently not present in HIG preparations [19,23,66]. HCMV infection inhibits TBPC self-renewal, proliferation and differentiation, which are required for proper villous development [37]. High affinity, potent human mAbs to gB, which functions in virion entry into a broad range of cell types, should be considered as a potential biotherapy for congenital HCMV infection, alone or in combination with pentamer complex targeting mAbs that more efficiently block infection of epithelial and endothelial cells. Additional advantages include a well-defined composition that reduces lot-dependent effects and increases consistency, suggesting mAbs could have considerable efficacy in clinical trials.

## 3. Experimental Section

### 3.1. Cells

TBPCs (from 7.3 and 15.6 weeks of gestation) were established as reported [27]. Cells were grown on gelatin-coated plates in DMEM/F12 supplemented with 10 ng/mL basic FGF (R&D Systems, Minneapolis, MN, USA), 10% FCS, 10 µM SB431542 (Tocris Bioscience, Minneapolis, MN, USA), 100 units/mL penicillin, 100 µg/mL streptomycin and 0.25 µg/mL fungizone. To exclude the presence of placental fibroblasts in the TBPC cultures, cells were immunostained for cytokeratin 7 (CK7), GATA-3 and GATA-4 as described in Section 3.3. These proteins are expressed in TBPCs but not in placental fibroblasts. ARPE-19 cells and HFFs were cultured in DMEM supplemented with FCS and penicillin/streptomycin as described previously [67,68].

### 3.2. Viruses and Infections

VR1814 (an endothelial and epithelial cell-tropic pathogenic strain of HCMV) [69] was propagated in HUVEC [70], followed by a single passage in HFFs to obtain high titer stocks. AD169 (attenuated laboratory strain), TB40/E-derived parental virus (vBAC4-luc) and ΔUL131A mutant (vBAC4-luc/UL131Astop) were propagated in HFFs [53]. For infection (MOI is indicated in figure legends), virus was diluted in DMEM, 1% FCS, 100 units/mL penicillin, 100 µg/mL streptomycin and adsorbed for 2 h. Virus suspensions were aspirated, cells washed with PBS and provided with fresh medium.

### 3.3. Immunofluorescence

Cells were fixed with 4% paraformaldehyde, permeabilized with 0.5% Triton X-100 and blocked with normal serum. Cells were incubated with primary antibody, followed by fluorescein isothiocyanate (FITC) or rhodamine red-X (RRX) labeled secondary antibodies (Jackson ImmunoResearch Laboratories, West Grove, PA, USA). For detection of TBPC markers, antibodies to cytokeratin 7 (CK7, monoclonal rat IgG, clone 7D3 [71]), GATA-3 (polyclonal goat IgG, R&D Systems, Minneapolis, MN, USA), GATA-4 (polyclonal goat IgG, R&D Systems) and HMGA2 (polyclonal rabbit IgG, Abcam, Cambridge, MA, USA) were used. Murine monoclonal antibodies used to detect viral proteins were CH433 (anti-IE1, immediate-early protein), CH19 (anti-pp28, true-late protein) [72] and M23 (anti-pUL112/pUL113, early proteins) [73]. Nuclei were counterstained with DAPI (Sigma-Aldrich, St. Louis, MO, USA). Analyses were performed with a Nikon Eclipse TS100 inverted fluorescence microscope equipped with a Nikon DS-F12 camera controlled by Nikon NIS Elements 4.0 imaging software [74].

### 3.4. Detection and Quantification of Viral Progeny

To quantify viral progeny, TBPCs or HFFs were infected with VR1814 or AD169 (MOI 0.1). Conditioned media (CM) was collected every 48 h. Subsequently, virus titers in CM were determined with an immunofluorescence-based infectivity assay in HFFs at 24 hpi [70]. For quantification, at least six representative pictures were taken and IE1-positive cells counted using Fiji Image J software [75,76].

### 3.5. Virus Neutralization Assay

HCMV neutralizing assays were performed as described before [36]. Briefly, TBPCs or ARPE-19 cells were seeded in 24-well plates. Diluted mAbs (final concentration 0.01 µg/mL, 0.1 µg/mL, 1 µg/mL or 10 µg/mL) or HIG (Cytotect, Biotest, Boca Raton, FL, USA, final concentration 0.1 µg/mL, 1 µg/mL, 10 µg/mL or 100 µg/mL) were incubated with VR1814 (MOI 0.01, in 300 µL total volume) for 1 h at 37 °C with moderate agitation. Virus-antibody mixtures were adsorbed to cells for 2 h, followed by a wash step with PBS. The cells were then supplemented with fresh growth medium. At 2 dpi cells were fixed, permeabilized and immunostained for IE1 and analyzed by immunofluorescence microscopy (Section 3.3). IE1-positive cells were quantified as described in Section 3.4. Alternatively, viral titers were adjusted to give a total of 600 to 800 IE1-positive cells/well in 24 well plates. To target gB, human mAb TRL345 (Trellis Bioscience, LLC, South San Francisco, NC, USA [23]) was used. To target the pentamer, human mAb 1F11 or 5A2 (cloned from a published sequence [19] by Trellis Bioscience) directed to pUL130/pUL131A was used. Synagis [77], reactive with respiratory syncytial virus, was used as negative control mAb. 

### 3.6. Trophosphere Formation

TBPCs were mock infected or infected with VR1814 or a mixture of virus and mAb as described above. The corresponding mAb used for pretreatment was present throughout the experiment (20 µg/mL). At 3 dpi, TBPCs were dissociated and reseeded in matrigel-coated plates (100,000 cells per 2 cm^2^) in differentiation medium (Knockout Serum Replacement Medium, Gibco, Grand Island, NE, USA) supplemented with 10% FCS, 100 units/mL penicillin, 100 µg/mL streptomycin, 0.25 µg/mL fungizone, 10 ng/mL FGF4 (Sigma-Aldrich, St. Louis, MO, USA) and 40 ng/mL EGF (Invitrogen, Grand Island, NE, USA) [27]. Formation of trophospheres was analyzed by microscopy at 4 dpi.

## 4. Conclusions

Human TBPCs in the chorion differentiate into CTBs and STBs, the two major cell types of chorionic villi required for placental development. The HCMV pathogenic strain VR1814 and the attenuated strain AD169 replicate fully in TBPCs, and the infectious progeny released retain tropism. Infection is blocked by a human mAb TRL345 to gB but not mAbs to the pentamer complex. Infected TBPCs are functionally impaired and fail to develop into large trophospheres comprised of cells that express the properties of differentiated CTBs and STBs. Altogether, our results suggest that congenital HCMV infection could be suppressed with a broadly effective neutralizing mAb to gB to prevent infection of TBPCs and, together with mAbs that protect specialized placental cells, could reduce virus transmission at the uterine-placental interface. 
